# Evaluating the efficacy and safety of electro-acupuncture in patients with antipsychotic-related constipation: protocol for a randomized controlled trial

**DOI:** 10.1186/s13063-021-05732-5

**Published:** 2021-11-04

**Authors:** Fang-Ting Yu, Di-He Long, Guang-Xia Shi, Li-Qiong Wang, Jian-Feng Tu, Li-Li Gang, Fu-Quan Liu, Yang Wang, Xiao Cui, Si Bao, Yu Yu, Wei Wei, Shi-Yan Yan, Jing-Wen Yang, Cun-Zhi Liu

**Affiliations:** 1 International Acupuncture and Moxibustion Innovation Institute, School of Acupuncture-Moxibustion and Tuina, Beijing University of Chinese Medicine, Beijing, 100029 China; 2 Beijing Changping Hospital of Integrated Chinese and Western Medicine, Beijing, 102208 China

**Keywords:** Electro-acupuncture, Antipsychotic-related constipation, Randomized controlled trial

## Abstract

**Background:**

Constipation is known as a common adverse effect of antipsychotics. Advice for its management remains inadequate. This study is designed to investigate the efficacy and safety of electro-acupuncture (EA) for antipsychotic-related constipation.

**Methods:**

This is a single-centric, parallel-group, randomized controlled trial with blinded participants, outcome assessor, and statistician. One hundred twelve participants will be randomly assigned into the EA group or sham acupuncture (SA) group in a 1:1 ratio. The study will last for 22 weeks for each participant, including a 2-week baseline assessment period, an 8-week treatment period, and a follow-up for 12 weeks. The primary outcome is the change of mean weekly complete spontaneous bowel movements (CSBMs) during weeks 1 to 8 from baseline. Secondary outcomes include the change from baseline of mean weekly CSBMs during the follow-up period, mean weekly spontaneous bowel movements (SBMs), overall CSBM response rate, scores on Bristol Stool Form Scale (BSFS), straining level, Patient Assessment of Constipation Symptoms (PAC-SYM), Patient Assessment of Constipation Quality of life questionnaire (PAC-QOL), and Brief Psychiatric Rating Scale (BPRS). Adverse events and medicine use will be recorded as well.

**Discussion:**

The study is designed based on a rigorous methodology to evaluate the efficacy and safety of EA for antipsychotic-related constipation. The finding will be published in peer-reviewed journals as reliable evidence.

**Trial registration:**

ClinicalTrials.gov ChiCTR2000032582. Registered May 3, 2020, with the Chinese Clinical Trial Registry.

**Supplementary Information:**

The online version contains supplementary material available at 10.1186/s13063-021-05732-5.

## Background

Antipsychotics are the cornerstone for treating schizophrenia [1, 2]. They play an irreplaceable role in relieving hallucinations and improve disorganized behaviors [3]. However, a multitude of unrecognized adverse effects usually hampers the utility as a disturbing problem. Antipsychotic-related constipation is one well-known adverse effect that frequently occurs in one-third of psychiatric patients taking antipsychotics [4, 5]. It negatively impacts life quality and may develop into severe gastrointestinal conditions (e.g., paralytic ileus and intestine perforation) [6, 7].

High-quality evidence for managing antipsychotic-related constipation remains scarce [8]. In clinical practice, laxatives are commonly prescribed next to lifestyle and dietary modifications [3]. However, despite side effects as diarrhea and abdominal pain [9], laxatives produce only short-term effects and patients tend to relapse [10, 11]. Because of efficacy and safety concerns, nearly half of constipated patients were dissatisfied with traditional treatment [12]. Effective nonpharmacological treatments may be potential options.

Electro-acupuncture (EA) is the technique to provide enhanced treatment effect with a low-intensity electric current connected to acupuncture needles. It has been widely used for treating gastric disorders in clinical practice as a potentially effective therapy. Recent evidence indicated that acupuncture was noninferior to prucalopride for functional constipation with fewer adverse effects [13]. Considering the particularity of schizophrenic patients, whether acupuncture is efficient for treating antipsychotic-related constipation remains unknown. This randomized controlled trial (RCT) is designed to investigate the efficacy and safety of electro-acupuncture for antipsychotic-related constipation. Our hypothesis is that the efficacy of EA is superior to sham acupuncture (SA) and should be safe and tolerable for patients with antipsychotic-related constipation.

## Methods/design

### Study design

**Fig. 1:**
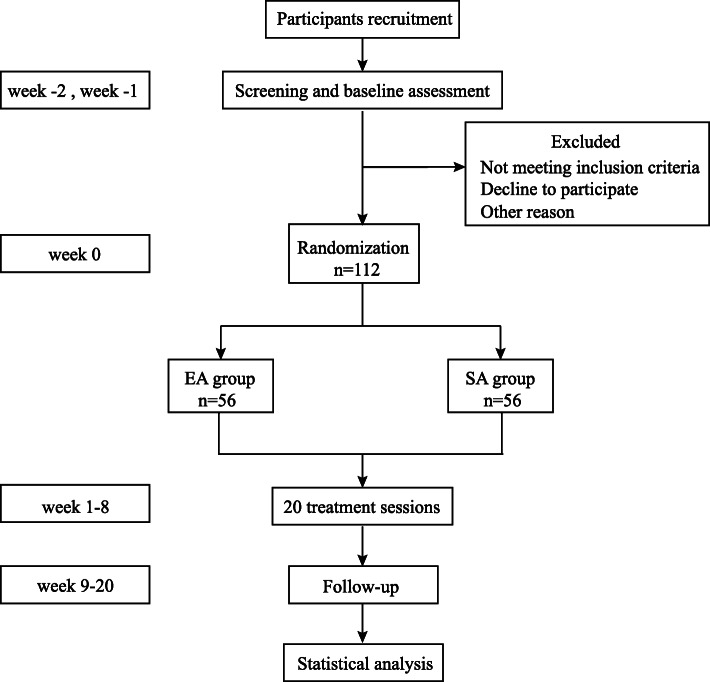
Flow diagram of study design. Abbreviation: EA, electro-acupuncture; SA, sham acupuncture

This is a prospective, single-centric, randomized controlled trial with two parallel groups randomized at a 1:1 allocation. It is designed following the Consolidated Standards of Reporting Trials (CONSORT) and the Standards for Reporting Interventions in Clinical Trials of Acupuncture (STRICTA) guidelines [14, 15], and is reported based on the Standard Protocol Items (SPIRIT) [16]. The study has been registered at the Chinese Clinical Trial Registry (http://www.chictr.org.cn) with the registration number ChiCTR2000032582. The Ethics Committee of Beijing Changping Hospital of Integrated Chinese and Western Medicine has approved the study protocol (No. 2019-5). A detailed study procedure is shown in Fig. 1.

#### Study setting

The study will be conducted at the Beijing Changping Hospital of Integrated Chinese and Western Medicine which is known for its psychiatric treatment accommodating a thousand mental inpatients.

### Participants

#### Recruitment

Psychiatric patients who are hospitalized at the Beijing Changping Hospital of Integrated Chinese and Western Medicine will be recruited for this study. Clinicians will initially screen and identify the patients who are interested in participation. Researchers will confirm the eligibility and written informed consent will be obtained from each participant subsequently. To improve recruitment and retention, post-trial acupuncture treatment will be offered for participants completing the entire study.

#### Diagnose criteria

Diagnosis of schizophrenia will be confirmed by two attending psychiatrists according to the Diagnostic and Statistical Manual of Mental Disorders, fifth edition (DSM-5) using the Structured Clinical Interview for DSM-5, Clinical Trials Version (SCID-5-CT) [17, 18]. Antipsychotic-related constipation is defined as<3 spontaneous bowel movements (SBMs) per week with ≥1 of the following symptoms in at least 25% of bowel movements during the baseline assessment period: lumpy or hard stools based on the Bristol Stool Form Scale (BSFS) [19], straining, sensation of incomplete evacuation, or anorectal obstruction induced by antipsychotics. Bowel movements will be considered spontaneous when no assistant methods are used in the previous 24 h.

#### Inclusion criteria

Patients will be included if they meet the following criteria: (1) aged between 18 and 65 years, (2) meeting the diagnoses of schizophrenia and antipsychotic-related constipation, (3) taking antipsychotic drugs over the last 3 months and will maintain the use, (4) having<3 complete SBMs (CSBMs) per week characterized by a complete sense within a baseline assessment period, and (5) available to sign informed consent.

#### Exclusion criteria

Exclusion criteria include that (1) constipation caused by other reasons than antipsychotic drugs; (2) mental instability within baseline assessment period judged by clinicians; (3) severe heart, liver, kidney diseases, or any other considerable dysfunctions (e.g., gastrointestinal obstruction); (4) use of anticholinergic drugs or drugs for constipation (except for rescue medicine) within 2 weeks before randomization; (5) pregnant or breastfeeding women; (6) participants with a pacemaker, metal allergy, or severe fear of needling; (7) a history of acupuncture treatment within the past 3 months; and (8) participation in any other clinical trials meanwhile.

### Randomization

According to the allocation schedule, participants will be randomly assigned into the EA group or SA group in a 1:1 ratio. An independent statistician unrelated to the final statistical analysis will generate a randomization sequence using SPSS Statistics, version 21.0 (International Business Machines Corporation, China). Numbered opaque envelopes that containing allocation codes will remain sealed until the first treatment. An independent researcher is available to open the corresponding envelope and complete the allocation. The assigned result will be informed to the acupuncturist only.

### Blinding

Participants, outcome assessors, and statisticians will be blinded in this trial. Given the nature of the intervention, the acupuncturist will not be blinded. Participants will remain in a supine position to receive treatment with no visual information about the type of intervention. Outcome assessors will complete evaluations before acupuncture treatment for that week to remain blinded. The statistician will perform analyses without knowing the group information. To ensure the overall quality, it is not permissible to reveal the allocated intervention to the participants, the assessors, or the statistician during this study.

### Interventions

Twenty sessions of 30-min treatment (3 sessions per week during the first 4 weeks and 2 sessions per week during the last 4 weeks) will be provided for all participants over 8 weeks. Acupoints for antipsychotic-related constipation were predefined by acupuncture experts according to the TCM theory. Licensed acupuncturists with experience of at least 3 years will offer the treatments required. Besides, all enrolled participants will receive a basic treatment of schizophrenia according to the Guideline for Prevention and Treatment of Schizophrenia, the second edition developed by the Psychiatric Branch of the Chinese Medical Association [20]. Diet and lifestyle will be similar among participants and medicine use will be supervised by nurses. Participants may discontinue their intervention if they have considerable conditions such as worsening constipation or mental instability, judged by assessors or clinicians. Participants with no bowel movements for at least 3 consecutive days will be allowed to use 5–10 mg enteric-coated bisacodyl or 110-ml glycerol as rescue medicines. Additional treatment for constipation will be avoided as possible during this study.

#### EA group

Semi-standardized treatment with 5 obligatory acupoints and 1 adjunct acupoint will be offered. All acupoints are localized based on the WHO Standard Acupuncture shown in Table 1 [21]. In this group, obligatory acupoints consist of bilateral Tianshu (ST25), Fujie (SP14), Quchi (LI11), Zhigou (TE6), and Shangjuxu (ST37). According to the syndrome differentiation of Traditional Chinese Medicine (TCM) [22, 23], bilateral Hegu (LI4) will be added for participants with heat patterns (e.g., dry mouth, surging pulse, or a red tongue) as adjunct acupoint. Similarly, bilateral Taichong (LR3) and bilateral Zulanli (ST36) will be added for Qi stagnation pattern (e.g., bloating, loss of appetite, or frequent burp) and deficient pattern (e.g., fatigue, pale tongue, or a thread weak pulse), respectively.

**Table 1:** Location of acupoints in electro-acupuncture group

Acupoints		Location
Obligatory acupoints	Tianshu (ST25)	On the upper abdomen, 2 cun lateral to the center of the umbilicus
	Fujie (SP14)	On the lower abdomen, 1.3 cun inferior to the center of the umbilicus, 4 cun lateral to the anterior median line
	Quchi (LI11)	On the lateral aspect of the elbow, at the midpoint of the line connecting LU5 with the lateral epicondyle of the humerus
	Zhigou (TE6)	On the posterior aspect of the forearm, midpoint of the interosseous space between the radius and the ulna, 3 cun proximal to the dorsal wrist crease
	Shangjuxu (ST37)	On the anterior aspect of the leg, on the line connecting ST35 with ST41, 6 cun inferior to ST35
Adjunct acupoint for heat pattern	Hegu (LI4)	On the dorsum of the hand, radial to the midpoint of the second metacarpal bone
Adjunct acupoint for Qi stagnation pattern	Taichong (LR3)	On the dorsum of the foot, between the first and second metatarsal bones, in the depression distal to the junction of the bases of the two bones, over the dorsalis pedis artery
Adjunct acupoint for deficient pattern	Zusanli (ST36)	On the anterior aspect of the leg, on the line connecting ST35 with ST41, 3 cun inferior to ST35

One “cun” is defined as the width of the interphalangeal joint of patient’s thumb

When in a supine position, strict disinfection with 75% alcohol will be implemented on participants’ skin around acupoints. Disposable stainless steel needles (0.30×50/75mm, Suzhou Huatuo Medical Instrument Co., Ltd.) will be inserted in ST25 and SP14 to approximately 30–70mm. Paired alligator clips from an EA apparatus (Suzhou Huatuo Medical Instrument Co., Ltd.) will then be attached to needle holders at bilateral ST25 and SP14. Current stimulation will be put subsequently with a dilatational wave of 10/50 Hz and intensity from 0.1 to 1 mA (skin vibrate mildly without pain). Other acupoints will be treated with needles (0.25×40mm) inserted to about 20–30 mm along with manipulations of twirling, lifting, and thrusting. The achievement of compositional sensations including soreness, numbness, distention, or heaviness, known as de qi, is indicative of effective needling.

#### SA group

Non-acupoints (Table 2) close to acupoints in the EA group will be treated. Superficial skin penetration (depth of 2–3mm) will be performed by 0.25×25mm needles with no manipulations. Similar to the EA group, paired alligator clips will be attached to needle holders at bilateral sham ST25 and sham SP14 while no flow of electric current will be outputted. Other treatment settings are identical to those in the EA group.

**Table 2:** Location of non-acupoints in sham acupuncture group

Acupoints		Location
Obligatory non-acupoints	Sham Tianshu	On the upper abdomen, 1 inch beside ST25, between the Spleen Meridian and the Stomach Meridian
	Sham Fujie	On the lower abdomen, 1 inch beside SP14, between the Spleen Meridian and the Stomach Meridian
	Sham Quchi	On the lateral aspect of the elbow, at the midpoint between the Large Intestine Meridian and the Lung Meridian beside LI11
	Sham Zhigou	On the posterior aspect of the forearm, 6 cun proximal to the dorsal wrist crease, at the radial margin of the ulna
	Sham Shangjuxu	On the anterior aspect of the leg, at the midpoint between the Stomach Meridian and the Gallbladder Meridian beside ST37
Adjunct non-acupoint for heat pattern	Sham Hegu	On the dorsum of the hand, between the 3rd and 4th metacarpals, in the depression proximal to the 3rd metacarpophalangeal joint
Adjunct non-acupoint for Qi stagnation pattern	Sham Taichong	On the dorsum of the foot, between the third and fourth metatarsal bones, in the depression distal to the junction of the bases of the two bones
Adjunct non-acupoint for deficient pattern	Sham Zusanli	On the anterior aspect of the leg, 3 cun below GB34, between the Stomach Meridian and the Gallbladder Meridian

One “cun” is defined as the width of the interphalangeal joint of patient’s thumb

### Outcomes

#### Primary outcome

**Fig. 2:**
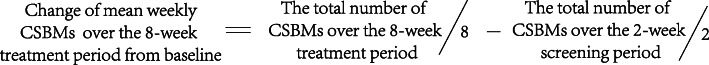
The calculating formula for primary outcome

**Fig. 3:**
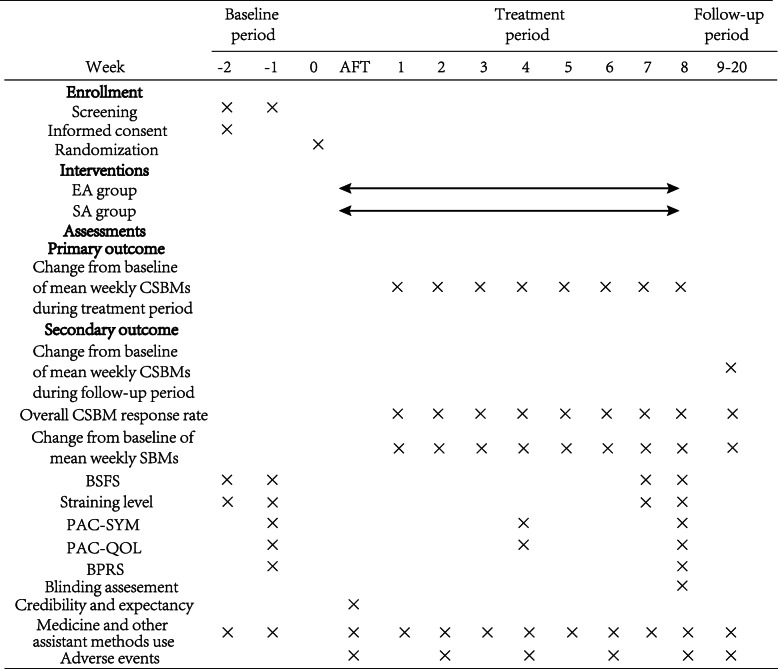
Standard Protocol Items: Recommendations for Interventional Trials (SPIRIT) and the schedule of the trial. Abbreviation: AFT, after the first treatment; EA, electro-acupuncture; SA, sham acupuncture; SBM, spontaneous bowel movement; CSBM, complete spontaneous bowel movement; BSFS, Bristol Stool Form Scale; PAC-SYM, Patient Assessment of Constipation Symptoms; PAC-QOL, Patient Assessment of Constipation Quality of life questionnaire; BPRS, Brief Psychiatric Rating Scale

The primary outcome is defined as the change of mean weekly CSBMs over the 8-week treatment period from baseline, which will be obtained from a stool diary (calculating formula shown in Fig. 2). The stool diary is regarded as a valid instrument to reflect bowel habits in constipated participants [24]. The caregivers for participants will be trained to complete stool diaries during the whole period of the study. Weekly defecation frequency, stool consistency (types 1–7 on BSFS), straining level (scores from 0 to 4 representing the difficulty of defecation increasingly), a complete sense of each bowel movement (yes or no), medicine use, or other assistant methods will be recorded. The Schedule of each outcome assessment is illustrated in Fig. 3.

#### Secondary outcomes

##### Change from baseline of mean weekly CSBMs during the follow-up period

Similar to the primary outcome, change from baseline of mean weekly CSBMs in the rest of three 4-week periods (from weeks 9 to 12, weeks 13 to 16, and weeks 17 to 20) will be calculated and compared between groups. The data will be used to verify whether there is a sustained effect by showing the extent.

##### Overall CSBM response rate

Participants are thought to be responders when they have ≥3 CSBMs and ≥1 CSBM increasing from baseline for at least 6 weeks of the treatment period [25]. If participants miss recording stool diaries for at least 4 of 7 days in a given week, they will not be considered as responders during that week. Participants who discontinue the study prematurely will be classified as non-responders for the rest of the weeks.

##### Change from baseline of mean weekly SBMs

Change from baseline of mean weekly SBMs during the 8-week treatment period and 12-week follow-up period will be assessed.

##### Bristol Stool Form Scale

The BSFS describes 7 stool types from hard lumps of stool to watery stool with aid images. Change of mean weekly scores on BSFS from baseline to the last 2 weeks of the treatment period will be compared between groups to detect a possible difference in stool consistency.

##### Straining level

The degree of difficulty during defecation will be recorded in stool diaries with scores ranging from 0 (no difficult) to 3 (too difficult that need assistant methods). Change of straining level from baseline to the last 2 weeks of treatment period will be compared.

##### Patient Assessment of Constipation Symptoms (PAC-SYM)

The PAC-SYM is commonly used to value the presence and severity of constipation on 3 subscales including abdominal symptoms, stool symptoms, and rectal symptoms [26]. A scoring range of 0 to 4 is derived for each item. The PAC-SYM will be measured at baseline, week 4, and week 8.

##### Patient Assessment of Constipation Quality of life questionnaire (PAC-QOL)

PAC-QOL is capable to reflect life quality related to constipation on four subscales by scoring 28 items including physical discomfort, psychosocial discomfort, worries and concerns, and satisfaction [27]. In each item, the score ranges from 0 to 4 with lower scores indicating greater impairment or dissatisfaction. PAC-QOL will be assessed at baseline, week 4, and week 8.

##### Brief Psychiatric Rating Scale (BPRS)

BPRS is designed as a convenient tool to evaluate the severity of schizophrenic states [28]. It contains 18 items rated on a 7-point Likert scale, from 1 representing not present to 7 representing extremely severe. The BPRS will be measured at baseline and week 8.

##### Blinding assessment

To test the success rate of blinding, all participants will be requested to guess their groups by choosing one of the 3 options including EA group, SA group, or unclear at week 8.

##### Credibility and expectancy

The credibility and expectancy will be evaluated through the Credibility/Expectancy Questionnaire after the first treatment [29].

##### Use of rescue medicine and other assistant methods

The use of rescue medicine and other assistant methods will be documented in stool diaries. The proportion and frequency of participants using rescue medicine or other assistant methods will be reported and compared between groups.

##### Adverse events

All adverse events will be clearly recorded, managed, and monitored during the study, which will be collected through (1) inquiries by outcome assessors after each treatment and at the time of recycling stool diaries (every 2 weeks during the treatment period and every 4 weeks during the follow-up period); (2) identification from laboratory values as reported by clinicians during hospitalization; and (3) self-report by participants at any time during this study. Possible treatment-related adverse events include local bleeding, hematoma, itching, and dizziness, etc. Serious adverse events (e.g., death, life-threatening condition; severe or permanent disability, prolonged hospitalization) will be reported within 24 h to the principal investigator. Adverse events will be managed by acupuncturists or clinicians at the Beijing Changping Hospital of Integrated Chinese and Western Medicine based on its association to the intervention. An independent data and safety monitor board (DSMB) comprises experts who have an intimate knowledge of constipation, schizophrenia, and methodology is established to surveil the safety.

### Data collecting method

All measurements in this study will be completed by either outcome assessors or caregivers. The stool diary will be maintained by caregivers who will be trained to ask standard questions, identify the stool types based on the Bristol Stool Form Scale, and make the record at bedtime (no bowel movement in a day) or within 5 min after each bowel movement. The diary will be recycled every 2 weeks during the treatment period, and every 4 weeks during the follow-up period. Data will be collected and transferred to the paper case report forms (CRFs). Participants deviating from the study protocol (e.g., discontinuing intervention) will be encouraged to complete the stool diary and the remaining assessments. No biological specimens will be collected in this study.

### Data management

When completing the whole study, double data entry will be conducted by two independent researchers using EpiData, version 3.1 for proofreading. The principal investigator will have full access to the final database of this study. Other investigators are limited to accessing the final data without permission from the principal investigator. The result of the study will be published in a peer-reviewed journal. Participants’ information will remain anonymous including name, age, and telephone number, etc. All data related to the trial will be saved for at least 5 years after publication. The researcher with the approved proposal will be permitted to access the data by contacting the corresponding authors.

### Quality control

To ensure the accuracy, consistency, and completeness of the data, the outcome assessor will be responsible for guiding, reminding, and supervising the record of the stool diary. Data of this study will be monitored primarily by the center leaders. The study designers from the Beijing University of Chinese Medicine will audit the study semi-annually to ensure the overall quality and the completeness of data.

### Sample size

Referring to a previous study [30], the change from baseline in mean weekly CSBMs during week 1 to week 8 is about 1.8 in the EA group and 0.9 in the SA group with a standard deviation of 1.6. A calculated sample size of 50 participants in each group is estimated based on a significant level of 0.05 and a statistical power of 0.8. When considering a dropout rate of 10%, 56 participants are needed in each group, 112 participants in total.

### Statistical analysis

A professional statistician who is blinded to the allocation will analyze data using IBM SPSS21.0. Continuous variables will be presented as mean (standard deviation) or the median (interquartile range) based on data distribution. Categorical variables will be described by frequencies and percentages. Statistical analysis will follow the intention-to-treat principle predominately for participants who have been randomized. Missing data for the primary outcome will be imputed using the multiple imputation method. The alternative hypothesis is that there is a significant difference between EA and SA groups in the change of mean weekly CSBMs over an 8-week treatment period from baseline.

For the primary outcome, analysis of covariance (ANCOVA) model will be used with treatment and corresponding baseline value as covariates. Repeated measures ANCOVA with the same covariates will be implemented for change from the baseline of mean weekly CSBMs during the follow-up period, and change from baseline of mean weekly SBMs. An independent *t* test or Wilcoxon rank-sum test will be implemented for scores on BSFS, straining level, BPRS, credibility and expectancy, and mean weekly frequency of rescue medicine and other assistant methods used. The difference in overall CSBM response rate, blinding, adverse events, and proportion of participants using rescue medicine or other assistant methods will be compared using the Pearson *χ*^2^ test or Fisher’s exact test. Repeated measures ANOVA or Kruskal-Wallis *H* test will be implemented for scores on PAC-SYM and PAC-QOL. All significance levels will be set at 0.05 (two-sided) with confidence intervals reported.

As sensitive analyses, a per-protocol analysis will exclude data from participants who complete less than 16 sessions (80% of total treatment sessions) or use other medicines to treat constipation for more than 3 days per week on average. No additional analyses will be performed.

## Discussion

Antipsychotic-related constipation is a common discomfort among psychiatric patients [4]. The lack of a uniform definition for antipsychotic-related constipation might limit relative researches. The diagnosis criteria in our study are made based on clinical indications and refer to the definition of constipation suggested by the American College of Gastroenterology Task Forces and the definition of opioid-induced constipation in Rome IV [31, 32]. This present study, to our knowledge, is the first RCT evaluating the efficacy of EA for antipsychotic-related constipation.

Currently, high-quality evidence of effective interventions for antipsychotic-related constipation remains scarce [8]. The latest small-scale study reported that prucalopride was more efficacious than lactulose for constipation induced by clozapine [33]. However, it is open-label with no random sequence generation performed. Well-designed RCTs for antipsychotic-related constipation are needed. One RCT investigated the efficacy of orlistat (a weight-control medication) for constipation induced by clozapine. While nearly half of the participants were reported with diarrhea that leads to discontinuation [34]. Acupuncture is regarded as a safe therapy with few side effects reported which might be convenient and beneficial for schizophrenic inpatients [35].

The strengths of this study include that it is designed based on a rigorous methodology. Participants will be screened for eligibility through a 2-week assessment period before randomization. Selections of acupoint are semi-standardized based on the syndrome differentiation of TCM which reflects practical conditions. Administration of diet, lifestyle, and medicine use will be conducted by nurses during the whole period of this study to reduce the potential impact. The study also has several limitations. First, a noninvasive acupuncture placebo might be difficult to use because participants might detect the feeling of no needling. We will use shallow needling for the SA group instead, which might produce an unexpected effect. Second, due to the nature of interventions, acupuncturists could not be blinded. To minimize the impact, they will be informed to limit unnecessary communications with participants. Third, the individualized treatment regimens for schizophrenia may present to be a potential confounding factor for analyses. Forth, it is a single-centric study and may limit generalizability in the future.

Whether acupuncture is effective for treating antipsychotic-induced constipation is unknown. We hope this study could provide reliable evidence and verify the potential value of acupuncture in this area.

### Trial status

Affected by the COVID-19 pandemic, there is no recruitment of this study (version 1.0, October 11, 2019) which is expected to start in December 2021 and be completed in June 2022.

## Supplementary Information

**Additional file 1..** SPIRIT 2013 Checklist: Recommended items to address in a clinical trial protocol and related documents. doc.

**Additional file 2..** Model informed consent.

## Data Availability

Not applicable.

## Abbreviations

EA: Electro-acupuncture
SA: Sham acupuncture
RCT: Randomized controlled trial
SBM: Spontaneous bowel movements
CSBM: Complete spontaneous bowel movement
BSFS: Bristol Stool Form Scale
PAC-SYM: Patient Assessment of Constipation Symptoms
PAC-QOL: Patient Assessment of Constipation Quality of life questionnaire
BPRS: Brief Psychiatric Rating Scale
CONSORT: Consolidated Standards of Reporting Trials
STRICTA: Standards for Reporting Interventions in Clinical Trials of Acupuncture
SPIRIT: Standard Protocol Items
DSM-5: Diagnostic and Statistical Manual of Mental Disorders, fifth edition
SCID-5-CT: Structured Clinical Interview for DSM-5, Clinical Trials Version
DSMB: Data and safety monitor board
CRF: Case report form
ANCOVA: Analysis of covariance
