# Supplementary material for: Evaluating the efficacy and safety of electro-acupuncture in patients with antipsychotic-related constipation: protocol for a randomized controlled trial
Source: Trials. 2021 Nov 4;22:771. doi: 10.1186/s13063-021-05732-5 (PMC8567121; doi:10.1186/s13063-021-05732-5)
Supplement: Supplementary file 2 — Additional file 2.. Model informed consent. [file 13063_2021_5732_MOESM2_ESM.docx]

**Informed Consent**

Dear patient:

If you were diagnosed with antipsychotic-related constipation by your doctor, we invite you to participate in this study aiming to evaluate the efficacy and safety of electro-acupuncture for managing antipsychotic-related constipation. You need to decide whether you want to participate or not. It is helpful for you to introduce about the study aim, procedures, benefits and risks of the study, inconvenience and potential

discomforts during the study. This study is supported and funded by the National Science Fund for Distinguished Young Scholars (No: 81825024). Please carefully read the document and feel free to ask the study doctor about any question which you may have. You may discuss with family or friends before making your decision who can better understand this study and your options.

**Why is this study being done?**

Antipsychotics are the cornerstone for treating schizophrenia. They play an irreplaceable role in relieving hallucinations and improve disorganized behaviors. However, a multitude of unrecognized adverse effects usually hamper the utility as a disturbing problem. Antipsychotic-related constipation is one well-known adverse effect that frequently occurs in one-third of psychiatric patients taking antipsychotics. It negatively impacts life quality and may develop into severe gastrointestinal conditions (e.g., paralytic ileus and intestine perforation).

**What is electro-acupuncture?**

Electro-acupuncture is the technique to provide enhanced treatment effect with low-intensity electric current connected to acupuncture needles. It has been widely used for treating gastric disorders in clinical practice, supported by the regulatory bodies in China, and is regarded as a potential effective and safe therapy.

**What is the aim of this study?**

This study is designed to investigate the efficacy and safety of electro-acupuncture for antipsychotic-related constipation.

**Which intervention can I received?**

When you decide to participate, you will be randomly assigned into either the electro-acupuncture group or the sham acupuncture group. There will be 20 sessions of treatment offered for participants in both groups over 8 weeks. Additional treatment for constipation will be avoid as possible during this study.

**How long is the duration and what is the number of participants in the study?**

The study will last for 22 weeks for each participant, including a 2-week baseline assessment period, an 8-week treatment period, and follow-up for 12 weeks. We plan to enroll a total of 112 patients for this study.

**Who should be enrolled in this study?**

*Patients will be included if they meet the following criteria:*

1. aged between 18 and 65 years;
2. meeting the diagnoses of schizophrenia and antipsychotic-related constipation;
3. taking antipsychotic drugs over the last 3 months and will maintain the use;
4. having＜3 complete SBMs (CSBMs) per week characterized by a complete sense within baseline assessment period;
5. available to sign informed consent.

*Patients will not be included in this study if they have the followings:*

1. constipation caused by other reasons than antipsychotic drugs;
2. mental instability within baseline assessment period judged by doctors;
3. severe heart, liver, kidney diseases, or any other considerable dysfunctions (e.g., gastrointestinal obstruction);
4. use of anticholinergic drugs or drugs for constipation (except for rescue medicine) within 2 weeks before randomization;
5. pregnant or breastfeeding women;
6. participants with a pacemaker, metal allergy, or severe fear of needling;
7. a history of acupuncture treatment within the past 3 months;
8. participation in any other clinical trials meanwhile.

**Administration of the stool diary**

During the whole period of this study, your caregivers should record your defecation everyday on a stool diary. They will be trained to ask standard questions, identify the stool types based on the Bristol Stool Form Scale, and make the record at bedtime (no bowel movement in a day) or within 3 minutes after each bowel movement. The diary will be recycled every two weeks during treatment period, and every 4 weeks during the follow-up period.

**What risk effects may happen to me by participating in the study?**

Electro-acupuncture is a relatively safe procedure and few side-effects have been reported. You may feel nausea, dizziness and fainting during or after the treatment. Bleeding, hematoma, and other phenomena may occur after treatment, but these phenomena will release and disappear after a few days of acupuncture treatment. If you receive sham acupuncture during the study, your symptoms or condition may become worse, stay the same or improve. If you have no bowel movements for at least 3 consecutive days, you will be allowed to use 5–10 mg enteric-coated bisacodyl or 110-ml glycerol as rescue medicines. If you feel any discomforts during the treatment, you need to tell your doctors about your conditions immediately and then the doctors will evaluate the condition and give you appropriate medical treatment.

**What benefits can I expect?**

You may benefit from a reduction in their symptoms. The information will be beneficial in the management of other patients with a similar condition in the future. If you decide to participate in the study, you will get the study treatment for free during the study period. Your being in the study could help advance medical research. When you complete the entire study with no obvious deviations from study protocol, you are available to receive post-trial acupuncture treatment based on your needs (up to 5 sessions).

**Can I refuse to be in the study?**

Whether you participate in this study or not is depending on your desire totally. You can choose not to take part in the study, or you can drop out at any time without your doctor permission during the study. If you quit the study, you will receive the standard treatment as other patients in your hospital.

**Can I withdraw from this study?**

You can withdraw from this study if you have a research-related injury from the study intervention, need a treatment not allowed in this study, or any other reasons. If you pretend to stop participation, you need to inform the study doctors and give the specific reason.

**Confidentiality and privacy**

All information about patients will be kept confidential by the research group members. Only the clinical research members who responsible for the study may have access to your medical records. Your name will not appear in any publication or report related to this study. We will make every effort to protect the privacy of your personal information.

**Compensation for research-related injury**

If you are injured as a result of the study intervention performed during your participation, please contact the study assessors or your doctors immediately. Necessary medical treatment will be provided to assist your recovery from the injury. You will be covered by indemnity for harm that associated with the intervention.

**How to acquire correlative information of the study?**

If we notice any new information that may affect your decision to continue participating in the study, the doctors will keep you informed.

If you have any questions related to the study, please contact to Dr * (Tel: ********).

If you have any questions related to your personal benefits, you can consult the Ethics

Committee of the Beijing Changping Hospital of Integrated Chinese and Western Medicine (Tel: ********).

**Participant’s Statement of Consent**

● I have read the Participant Information Sheet, or someone has read it to me in a language that I understand.

● I understand the purposes, procedures and risks of the research described in the project.

● I give permission for my doctors, caregivers, and hospitals to release information concerning my disease and treatment for the purposes of this study. I understand that such information will remain confidential.

● I have had an opportunity to ask questions and I am satisfied with the answers I have received.

● I freely agree to participate in this study as described and understand that I am free to withdraw at any time during the study without affecting my future health care.

● I understand that I will be given a signed copy of this document to keep.

I voluntarily agree to take part in this research study.

Participant’s Printed Name Participant’s Signature

Date/Time

Investigator’s Printed Name Investigator’s Signature

Date/Time

**A relative or caregiver:**

As a relative or caregiver, I attest that I have read this entire form. I had enough time to consider this information, had an opportunity to ask questions and have had these answered satisfactorily. I agree and give permission to participate in the study on behalf of the person named above. Moreover, I confirm that this subject was provided with this information according to his/her ability to understand and the subject does not object to participation in the study.

Relative or caregiver’s Printed Name Participant’s Signature

Relations with patient Date/Time
